# Novel Immunotherapies for Osteosarcoma

**DOI:** 10.3389/fonc.2022.830546

**Published:** 2022-04-01

**Authors:** Yubao Lu, Jiahe Zhang, Yutong Chen, Yuchen Kang, Zhipeng Liao, Yuanqi He, Cangyu Zhang

**Affiliations:** ^1^ Department of Spine Surgery, The Third Affiliated Hospital of Sun Yat-sen University, Guangzhou, China; ^2^ The Second Clinical Medical College, Lanzhou University, Lanzhou, China; ^3^ Department of Orthopaedics, Lanzhou University Second Hospital, Lanzhou, China

**Keywords:** immunotherapies, osteosarcoma, immune microenvironment, PD-1, CAR T, TCR T, TIL T, cancer vaccines

## Abstract

Osteosarcoma (OS) is the most common primary malignant bone sarcoma mainly affecting adolescents and young adults, which often progresses to pulmonary metastasis and leads to the death of OS patients. OS is characterized as a highly heterogeneous cancer type and the underlying pathologic mechanisms triggering tumor progress and metastasis are incompletely recognized. Surgery combined with neoadjuvant and postoperative chemotherapy has elevated 5-year survival to over 70% for patients with localized OS tumors, as opposed to only 20% of patients with recurrence and/or metastasis. Therefore, novel therapeutic strategies are needed to overcome the drawbacks of conventional treatments. Immunotherapy is gaining momentum for the treatment of OS with an increasing number of FDA-approved therapies for malignancies resistant to conventional therapies. Here, we review the OS tumor microenvironment and appraise the promising immunotherapies available in the management of OS.

## Introduction

Osteosarcoma (OS), a malignant neoplasm with high morbidity in adolescents, basically stems from primitive mesenchymal cells, and is mostly seen in the metaphysis of femur, tibia, humerus, and other long bones (1). In light of incomplete statistics, the global incidence of osteosarcoma is about 4.8 per million (2, 3). Given the current clinical treatment, although surgery combined with neoadjuvant and postoperative chemotherapy has enhanced the 5-year survival rate by more than 70% in patients with localized OS, this conventional treatment failed in OS patients with recurrence and/or metastasis except for 20% of patients (4, 5). To make the matter worse, affected by the limitation in OS diagnosis, it is often the case that patients are tested for metastasis at the time of initial diagnosis (6). In terms of safety, mainstream chemotherapy (methotrexate, doxorubicin, cisplatin, and ifosfamide) appears with inescapable side effects like leukopenia, thrombocytopenia (7), mucositis (8), ototoxicity, and nephrotoxicity (9). Thus a novel less toxic therapy is needed. In a sense, OS is not only a disaster for patients and their families, but also a huge loss for social development. These losses do not simply come from the expensive medical services (for OS diagnosis and treatment) paid for by patients and the government. In fact, from a deeper perspective, it is because young patients are too unwell to enter society in order to fuel social development by that time. These losses are far more assessable and quantified. Therefore, applying new effective treatment methods to prolong the life span of patients and improve their quality of life is an essential problem that needs to be solved urgently.

**Figure 1:**
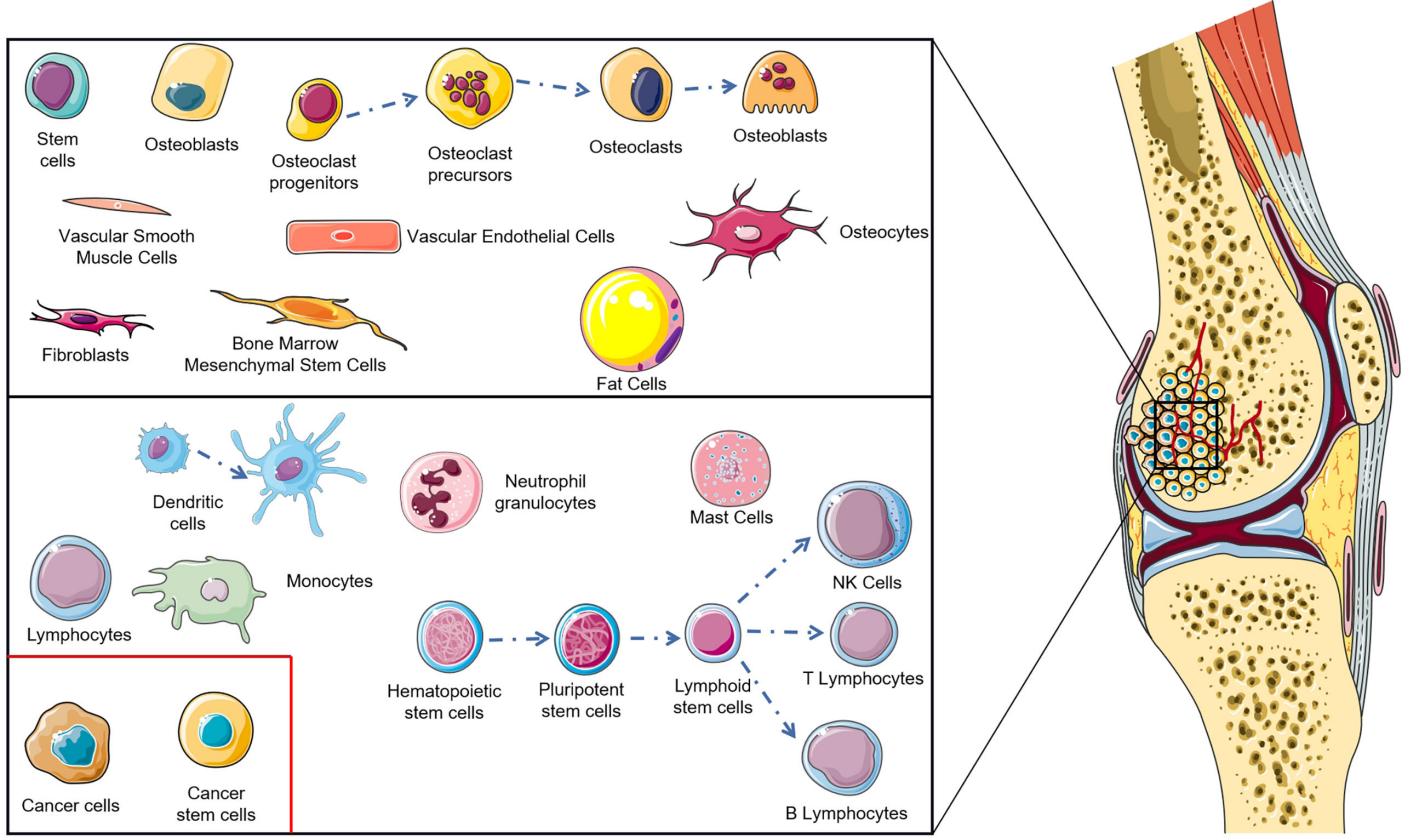
Cellular composition of osteosarcoma tumor microenvironment.

The tumor microenvironment is a key problem in modern cancer research, which has a decisive function in the occurrence and progression of tumors (10). The normal tissue microenvironment of bone consists of bone marrow stroma and mineralized extracellular matrix. Bone marrow contains two different cell types: hematopoietic stem cells and bone marrow-derived mesenchyme stem cells with hematopoietic function, which differentiate into non-blood cell components of bone, such as mesenchymal stem cells (MSCs), osteoblasts, osteoclasts, osteocytes, fibroblasts, adipocytes etc. (11–13). Benefitting from the particularity of the bone tissue microenvironment, the porous mineralized extracellular matrix structure and abundant nutrition supply enable the bone tissue microenvironment to become ‘fertilized soil’ for tumor growth (14, 15). During OS genesis, changes occur in the tissue microenvironment of bone, the most important of which lies in the infiltration of immune cells (macrophages, neutrophils, dendritic cells, mast cells, natural killer cells, T lymphocytes, and B lymphocytes) (**Figure 1**) (16). The imbalance of these factors in the OS localized microenvironment is considered as the key to modulate the progression and metastasis of OS. As research into the OS microenvironment deepens, immunotherapy has undergone rapid development. The monotherapy or combination therapy of a tumor vaccine, immunomodulator, genetically modified T cell, cytokine, and immune checkpoint inhibitor brings hope to the dilemma of OS treatment. This significant progress is attributed to the immunotherapy that avoids drug resistance and immune escape to a large extent, reducing side effects paralleling therapeutic effect improvement (17).

Hence, this review was carried out on immunotherapy in OS, aiming to provide systematic theoretical evidence for the development and application in the treatment, and give impetus to the progress of these kinds of studies.

## Tumor Immune Microenvironment (TME)

The network of the OS immune microenvironment is composed of both innate and adaptive immune cells, infiltrating with macrophages, neutrophils, dendritic cells (DCs), mast cells, natural killer (NK) cells, T lymphocytes, and B lymphocytes (18). Specifically, macrophages and T lymphocytes are the dominating constitution therein (19). These immune cells can be located in the core and invasive margin of the tumor or in the adjacent tertiary lymphoid structures (13). Indeed, a normal immune system can recognize tumor cells and initiate a series of functional stepwise events to eliminate them. This process is termed the cancer-immunity cycle and consists of seven major steps: 1) DCs detect neoantigens and pro-inflammatory cytokines released by tumor cells, 2) the captured antigens are presented to T cells, 3) the tumor-specific immunity is initiated with priming and activation of T-cell response, 4) T cells traffic to tumors and 5) infiltrate herein, 6) T cells identify and combine with tumor cells, and 7) kill them. Killed malignant cells release neoantigens and start a subsequent circulation (20). However, OS cells are able to modulate the recruitment and differentiation of immune cells, and consequently sabotage the cancer-immunity cycle and induce an immune-tolerant microenvironment which is conducive to proliferating and metastasizing tumor cells (21). Notably, OS patients with higher immune scores with enhanced immune cell infiltration in the microenvironment have better prognosis (22, 23). Therefore, the focus on the tumor immune microenvironment, especially its crosstalk with tumor cells, may be indispensable to further understand the OS immune system for developing novel immunotherapies.

### Tumor-Associated Macrophages in the TME

The immune component of OS TME predominantly consists of tumor-associated macrophages (TAMs), taking up a significantly high proportion compared with other immune cells (24), which play an essential role in inflammatory response and tissue homeostasis (25). Macrophages have high plasticity so that they present inverse phenotypes with different activating signals: the pro-inflammatory phenotype (M1) and the anti-inflammatory phenotype (M2) (26, 27). M1 macrophages serve as the key defense in tumor suppression by stimulating the immune system to express high levels of pro-inflammatory cytokines like IL-1, IL-6, and IL-12 (28), and both by inducting T helper type-1 (Th1) cell maturation and promoting inducible nitric-oxide synthase (iNOS) production (29). On the contrary, M2 macrophages are associated with immune suppression, matrix degrading, and tumor angiogenesis, and thus accelerate tumor progression and metastasis (27, 30). Many studies have proved that high infiltration of TAMs was correlated with worse prognosis in most solid cancers (31–33). Wolf-Dennen et al. and Li et al. revealed that M2-related cytokines, chemokines, and cell-markers showed an increased expression in lung metastasis of OS (34, 35). Similarly, Dhupkar et al. illustrated from another perspective that the shift from the M2 to M1 phenotype induced regression of lung metastasis in OS, strengthening the role of M2 macrophages in OS development (36). Nevertheless, it remains controversial to define whether TAMs are pro-tumor or anti-tumor in OS. Some previous studies showed that increased infiltration of TAMs was associated with reduced metastasis and a better survival outcome in high-grade OS (37, 38), whereas M2 macrophages indicated a bad prognosis (19, 38). Han et al. demonstrated that the number of M2 macrophages was negatively associated with TIM3^+^PD1^+^ T cells and relative pro-inflammatory cytokines (39). Through the CIBERSORT algorithm, M2 macrophages were verified as the major composition of TAMs. Buddingh et al. speculated that the constitutive of M2 macrophages may act in a metastatic suppression role rather than have a pro-metastatic effect in certain circumstances. M1 and M2 phenotypes are extremes of a continuum of macrophage polarization states (40). The extended transcriptional repertoire are detected from macrophages in chronic inflammation and tumors, which is mostly decided on the properties of diverse microenvironmental stimulation (41, 42). An intermediate phenotype M1-M2 found in primary OS with anti-metastatic activity suggested that the balance between M1 and M2 macrophages may play a decisive role in prognosis rather than the total number (38).

Therefore, M2-phenotype TAMs may work as promising targets to orchestrate OS progression. Novel treatment strategies focus on modulating TAM polarization to improve the ratio of M2 phenotype to M1 phenotype or inducing the shift from M2 phenotype to M1 phenotype to inhibit OS progression (**Table 1**). Mifamurtide, an immunomodulatory drug that triggers macrophages and increases the level of proinflammatory cytokines (56), has been approved by the European Medical Agency (EMA) to treat OS in combination with adjuvant chemotherapy (57, 58). Punzo et al. demonstrated mifamurtide’s anti-tumor effect from two aspects. Not only by shifting macrophage polarization towards the intermediate M1-M2 phenotype, it can also restrict M2 activation time *via* decreasing STAT3 and Akt phosphorylation to inhibit the STAT3 pathway and PI3K/Akt/mTOR pathway, both of which are activators of M2 polarization (43). In addition, all-trans retinoic acid (ATRA) has been proven to restrict the initiation of OS and reduce pulmonary OS metastasis (59, 60). ARTA could indirectly deviate macrophages from M2 polarization or disrupt the TAM-cancer stem cells (CSCs) pathway to limit CSC formation in OS (60). Naturally occurring compounds like dihydroxycoumarins (esculetin and fraxetin) were also expected to induce TAM polarization from M2 to M1 phenotypes in OS treatment (61). Dihydroxycoumarins act *via* inhibiting the production and growth of IL-10, MCP-1, and TGF-β_1_ as well as impeding the phosphorylation of Stat 3 during M2 phenotype differentiation to interfere with its activation (44, 61, 62). Zoledronic acid has been envisaged as a therapeutic drug because it is able to interfere with M2 phenotype polarization and cause TAM to polarize back to the M1 phenotype (45, 46). Although its function has been demonstrated in some bone metastasis tumors (63), a randomized study has suggested that zoledronate treatment resulted in higher risk than the placebo group (37). Gomez-Brouchet et al. found that zoledronate may induce deleterious polarization to CD68^+^/CD163^+^ bipotent macrophages (64). CD68 is stated to be an M1-polarized macrophage marker, while high level of CD163 staining is associated with high CMAF nuclear expression (a macrophage transcription factor correlated with M2 macrophage polarization) (65). Hence the effect of zoledronate needs more detailed research to clarify whether it is positive or negative for OS treatment.

**Table 1:** Therapeutic TAM targeting agents in OS.

Agent	Target cells and molecules	Mechanism	Reference
Mifamurtide	Monocytes and macrophages pSTAT3, pAKT, IL-17R TNF-α, IL-1, IL-6, IL-8, NO, PGE2, and PGD2 LFA-1, ICAM-1 and, HLA-DR	Switching TAM to polarize toward the intermediate M1-M2 phenotype	(32) Mifamurtide for the treatment of nonmetastatic osteosarcoma (33) Mifamurtide and TAM-like macrophages: effect on proliferation, migration, and differentiation of osteosarcoma cells
All-trans retinoic acid (ATRA)	CD117^+^Stro-1^+^ cells, cancer stem cells, and macrophages IL-1β, IL-4, IL-6, IL-13, and CXCL8	Decrease M2 phenotype polarization-induced stemness of OS	(34) All-trans retinoic acid prevents osteosarcoma metastasis by inhibiting M2 polarization of tumor-associated macrophages (35) Inhibition of M2-like macrophages by all-trans retinoic acid prevents cancer initiation and stemness in osteosarcoma cells
Dihydroxycoumarins (esculetin)	LM8 cells and macrophages Cyclin D_1_, CDK 4, MMP-2, TGF-β1, VEGF, IL-10, MCP-1, and pSTAT3	Downregulates the essential cytokines (TGF-β1, IL-10, and MCP-1) and protein (pSTAT3) in the differentiation of M2 macrophages	(36) Antitumor and antimetastatic actions of dihydroxycoumarins (esculetin or fraxetin) through the inhibition of M2 macrophage differentiation in tumor-associated macrophages and/or G1 arrest in tumor cells
Dihydroxycoumarins (fraxetin)	Macrophages IL-10, MCP-1, TGF-β1, and pSTAT3	Downregulates the essential cytokines (TGF-β1, IL-10, and MCP-1) and protein (pSTAT3) in the differentiation of M2 macrophages	(36) Antitumor and antimetastatic actions of dihydroxycoumarins (esculetin or fraxetin) through the inhibition of M2 macrophage differentiation in tumor-associated macrophages and/or G1 arrest in tumor cells
Zoledronic acid	Monocytes, dendritic cells, and macrophages IL-1b, TNF-α, VEGF, IL-10, IDO, IL-12, and polyI:C TGF-β, Arg-1, and Fizz-1	Upregulates M1-like cytokines Downregulates M2-like cytokines	(39) Zoledronic acid inhibits thyroid cancer stemness and metastasis by repressing M2-like tumor-associated macrophages-induced Wnt/β-catenin pathway (40) Zoledronic acid modulates antitumoral responses of prostate cancer-tumor-associated macrophages
Chimeric antigen receptor macrophage (CAR-M)	T cells, dendritic cells, and macrophages ERK and NF-κB(P65)	Upregulates pro-inflammatory pathways (interferon signaling, TH1 pathway, and iNOS signaling) in M2 macrophages	(43) Human chimeric antigen receptor macrophages Pluripotent stem cell-derived CAR-macrophage cells with antigen-dependent anti-cancer cell functions
Ferumoxytol (single or with CpG)	Monocytes and macrophages TNF-α, IL-12, IL-1α, IL-1β, IL-6, CD86, and iNOS	Enhances M1-like gene expression in TAMs	(44) Iron oxide nanoparticles inhibit tumour growth by inducing pro-inflammatory macrophage polarization in tumour tissues (45) Ferumoxytol and CpG oligodeoxynucleotide 2395 synergistically enhance antitumor activity of macrophages against NSCLC with EGFR L858R/T790M mutation
Porous hollow iron nanoparticle (PHNP)	Macrophages PI3K γ and NF-κB p65	Upregulates NF-κB p65 and downregulates PI3K γ in TAMs	(46) Polarization of tumor-associated macrophage phenotype *via* porous hollow iron nanoparticles for tumor immunotherapy *in vivo*

Burgeoning nanomaterials provide another possibility of modulating macrophage polarization in cancer (47–49). The Food and Drug Administration (FDA) has approved ferumoxytol applied in cancer treatment to polarize M2 macrophages to M1 and activate anti-tumor immune response in the TME (50, 51). Li et al. synthesized porous hollow iron oxide nanoparticles (PHNPs) loaded with PI3K γ inhibitor and successfully changed the phenotype of TAM (52). In this context, blocking M2-phenotype polarization signaling pathways such as PI3Kγ, ERK5-MAPK, and cMaf is a potential alternative for nanoparticle-based therapies (53–55).

### T Lymphocytes in the TME

T lymphocytes constitute the second most common infiltration cell type in OS. Particularly, tumor-infiltrating lymphocytes (TILs) are detected in 75% of OS with a peak around 86% in metastases, they include CD8^+^ T lymphocytes, CD4^+^ T lymphocytes, CD20^+^ B lymphocytes, and CD117^+^ mast cells (19). Helper and cytotoxic T cells can be activated by tumor antigen-triggered dendritic cells and directly attack tumors *via* cytotoxic cells (66). Besides, helper and cytotoxic T cells can also secret IFN-γ to inhibit tumor progression (67). In spite of a relatively low proportion of CD8^+^ T cell infiltration in OS, its ratio showed a significantly positive correlation with a lower rate of metastasis and better survival outcome (24). TILs are regarded as a selected population of T cells with higher specific immunological reactivity against tumors than normal lymphocytes. However, studies evaluated that CD8^+^ T cells were less abundant than myeloid cells in OS biopsies which suggests that OS has poorly immunogenic tumors with a lack of tumor neo-antigens (68). This would define OS as a “cold” tumor. Ligon et al. revealed that OS pulmonary metastasis was characterized with remarkable numbers of CD8^+^ T cells but the majority of them merely infiltrated on the edge of the metastasis because of immune resistance mechanisms (69). In addition, the expression of checkpoints TIM-3 and LAG-3 was detected on TILs at the interface of lung metastasis, indicating TIM-3 and LAG-3 are potential targets to enhanced TIL permeability and strengthen the cytotoxic effect (69).

On the basis of a data-driven mathematical model study, the population variation of cytotoxic T cells, helper T cells, and dendritic cells was in parallel with OS cell growth in the early stage and then decreased with time, and which was found to generally increase under the treatment of chemotherapy drugs as well (70). Whereas regulatory T cells were first reduced in the population and then raised (71). Fritzsching et al. showed that OS patients with a CD8^+^/FOXP3^+^ ratio above 3.08 possessed a notably improved survival, and the ratio of CD8^+^/FOXP3^+^ T cells to regulatory CD4^+^/FOXP3^+^ T cells in biopsies prior to chemotherapy allowed for the discrimination of OS patients with prolonged survival from non-survivors (68). On the other hand, the immune suppression molecule galectin-9 (Gal9) was primarily expressed on CD4^+^CD25^+^ Tregs in OS, with significantly higher frequencies than in non-cancer controls (72), as well as higher than other solid tumors (73). Furthermore, the Gal9 expressed by CD4^+^CD25^+^ Tregs could contribute to the development of M2 macrophages and lead to an increasingly suppressive anti-tumor CD8^+^ T cell response and inflammatory conditions (72). Another characteristic observed is the T cell exhaustion phenomenon that may be a vital mechanism contributing to impaired T-cell response against pathogens (74). The chronic inflammation in the TME can guide T cells to a dysfunctional or “exhausted” state, manifesting as an enhanced expression of multiple immune checkpoints of T cells (13). IL-21, mainly secreted from CD4^+^ T cells, has been proven to specifically enable homeostatic proliferation of cytotoxic CD8^+^ T cells by the effect of other cytokines such as IL-2, IL-7, and IL-15 (75, 76). Besides, it could also suppress the expression of FOXP3, a key transcription factor prompting Tregs, to relieve the inhibition on CD8^+^ T cell response (77). Gao et al. detected that CD4^+^ T cells in OS had decreased capacity to express IL-21 compared to healthy controls, especially follicular helper T (Tfh) cells which usually highly express PD-L1 with a severe reduction in this capacity as well as in proliferation capacity (78). Hence this evidence indicated that reverting the IL-21 decrease by blocking PD-L1 expression on Tfh cells or inhibiting naïve CD4^+^ T cell recruitment into tumors by interfering with PITPNM3 recognition of CCL18 may be an attractive strategy for immunotherapy in OS (24). IL-2 plays a crucial role in motivating expansion, differentiation, and function of effector T cells, and the loss or downward responsiveness of IL-2 are responsible for the exhausted phenotype in anti-tumor immunity (79). Tregs can be phenotypically identified by CD25 (IL-2 receptor-α subunit) (80). Remarkably, Solomon et al. developed Treg-depleting agents (RG6292) that targeted CD25, but did not interfere with IL-2 signaling on effector T cells (81). This breakthrough managed to reverse the suppressive states in the TME and preserved more available IL-2 to motivate effector T cells in the meanwhile.

### Immune-Related Cells

There exist some non-immune cells capable of effecting tumor ontogeny by mediating immune responses. Mesenchymal stem cells (MSCs) derived from both normal tissue and tumor tissue are confirmed to facilitate the progression of OS. The tumor promotional effect of MSCs can be attributed to two main mechanisms. One is capacitating OS cells with stemness properties by IL-6 secreted from MSCs (82). OS extracellular vesicles can cause MSCs to shift to the pro-tumor phenotype distinguished by abundant IL-6 production (83). The other is immunosuppression of activated MSCs, including inhibiting the proliferation of T cells (84), B cells (85), and NK cells (86), leading to the differentiation of Tregs (87).

Bone matrix remodeling is a distinct characteristic of osteosarcoma which is basically mediated by osteoclasts. Osteoclasts derive from monocytic lineage with high heterogeneity. Just like other immune cells with monocytic lineages (monocytes, macrophages, and dendritic cells, etc.), osteoclasts are qualified to regulate T-cell activation (88). The role of osteoclasts is determined by their precursor cells and microenvironment. In malignancies, they suppress the T-cell-mediated cytotoxicity of CD4^+^ and CD8^+^ cells to shelter tumor cells from immune elimination (89). The role of osteoclasts in the pathogenesis of OS remains controversial. Based on previous research, a cogent hypothesis suggests that osteoclasts promote tumor cell spread in the early stage, yet destruct and reorganize bone niches in the later stage (19). Generally, it is indispensable that osteoclasts are crucial regulators of OS growth through their flexible immune function. As in neoadjuvant chemotherapy, the macrophage-phagocyte system phagocytizes drug-carrier particles and then recruits them into bone tissues. Hence the osteoclasts, normally stemmed from macrophages in the bone microenvironment, in the vicinity of OS can represent the viability of macrophages and drug-delivered volume into bone marrow (90).

## Promising Immunotherapies

### Immune Checkpoint Inhibitors (ICIs)

In most types of cancer, two immune checkpoints, programmed cell death protein 1 (PD-1) (91, 92) and cytotoxic T lymphocyte-associated protein 4 (CTLA-4) (93, 94), were observed with an upregulated expression on T cells in the TME. Their activation capacitates immune tolerance and treatment resistance through inhibiting T-cell activation (95). Whereas, OS cells specifically adopt this deregulation mechanism with highly expressed ligand proteins that activate those pathways (96, 97). Consequently, by prohibiting engagement between infiltrated T cells and tumor cells, ICIs are introduced to reverse immune tolerance and elicit anti-tumor immune response. The 2018 Nobel Prize in Physiology and Medicine was awarded to Allison and Honjo for elucidating the immunosuppression of CTLA-4 and PD-1, respectively, and suggesting potential immunotherapy targeted at specific immune checkpoints. As two of the earliest studied immune checkpoints, some molecular mechanisms of PD-1 and CTLA-4 blockade have been clarified in the published research, like eliciting CD8^+^ T-cell and ICOS^+^ Th1-like cell infiltration in the TME (98). Moreover, with the combined therapy of PD-1 blockade and radiotherapy, CD8^+^ T cells can be stimulated at a great extent and trafficked to the distant metastatic lesions which is identified as an abscopal effect (99). In view of their encouraging results in anti-tumor function, ICIs involved with CTLA-4 inhibitors (ipilimumab), PD-1 inhibitors (nivolumab, pembrolizumab, and cemiplimab), and PD-L1 inhibitors (atezolizumab, avelumab, and durvalumab) have been approved by the FDA to treat numerous types of solid tumors: Hodgkin and non-Hodgkin lymphomas, melanoma, Merkel cell carcinoma, and liver, kidney, cervical, head and neck, lung, gastric, colorectal, and bladder cancers, and cutaneous squamous cell carcinoma (100).

#### PD-1/PD-L1 Inhibitor and CTLA-4 Inhibitor

ICIs targeting CTLA-4, PD-1, and PD-L1 were used as initial generation ICIs in the treatment of OS (**Table 2**). CTLA-4 is a transmembrane glycoprotein receptor expressed on Tregs and memory T cells, which suppresses anti-tumor immunity by engaging CD80/86 on DCs. Similarly, PD-1 is a transmembrane immunoglobulin widely distrusted on activated T cells, which functions as a CTL inhibitor and Tregs activator (101). As for PD-L1, the expression is detected on OS cells, which symbolizes reduced immune cell infiltration, including T cells, NK cells, and dendritic cells, as well as increased T-cell apoptosis (102). These inhibitors are capable of reinvigorating the T-cell-mediated anti-tumor immunity.

**Table 2:** Current PD-1/PD-L1 or CTLA-4-targeted ICI therapy.

Agent	Target	Subject	Therapeutic efficacy	Reference
Pembrolizumab	PD-1 inhibitor	Patients (≥18y) with recurrent or progressed OS	Well-tolerated but limited antitumor activity	(91) Pembrolizumab in advanced osteosarcoma: results of a single−arm, open−label, phase 2 trial
Nivolumab	PD-1 inhibitor	Patients (≤18y) with recurrent or refractory OS	Well-tolerated but no objective responses were observed in children	(78) Nivolumab in children and young adults with relapsed or refractory solid tumours or lymphoma (ADVL1412): a multicentre, open-label, single-arm, phase 1–2 trial
Atezolizumab	PD-L1 inhibitor	Patients (<30y) with progressive OS	Well-tolerated but limited response in all ages	(92) Atezolizumab for children and young adults with previously treated solid tumours, non-Hodgkin lymphoma, and Hodgkin lymphoma (iMATRIX): a multicentre phase 1–2 study
Apatinib+ camrelizumab	PD-1 inhibitor+ tyrosine kinase inhibitor	Patients (≥11y) with metastatic or locally advanced OS	PFS prolonged below expectation and AEs increased compared with single agent apatinib	(77) Apatinib plus camrelizumab (anti-PD1 therapy, SHR-1210) for advanced osteosarcoma (APFAO) progressing after chemotherapy: a single-arm, open-label, phase 2 trial
PD-1/CTLA-4 antibody+ Bempegaldesleukin	PD-1/CTLA-4 inhibitor+ CD122-preferential IL-2 pathway agonist	Disseminated K7M2-WT metastatic osteosarcoma mouse model, K7M3 primary tibial osteosarcoma mouse model, and DLM8 subcutaneous osteosarcoma mouse model	Durable tumor growth control with long-term survival, including complete cures	(93) Bempegaldesleukin (BEMPEG; NKTR-214) efficacy as a single agent and in combination with checkpoint-inhibitor therapy in mouse models of osteosarcoma
Nivolumab+ ipilimumab	PD-1 inhibitor+ CTLA-4 inhibitor	Patients (≥18y) with locally advanced, unresectable, or metastatic OS	Met predefined endpoint with manageable safety and high ORR	(96) Nivolumab with or without ipilimumab treatment for metastatic sarcoma (Alliance A091401): two open-label, non-comparative, randomized, phase 2 trials
PD-1/PD-L1 agent (pembrolizumab, nivolumab, Atezolizumab, or other)+ prednisone	PD-1 inhibitor+ steroid	Patients (≥21y) with advanced cancer (NSCLS, melanoma, renal cell carcinoma, and others)	Worsened ORR, PFS, and OS	(100) Integrated analysis of concomitant medications and oncological outcomes from PD-1/PD-L1 checkpoint inhibitors in clinical practice
Nivolumab+ tocilizumab	PD-1 inhibitor+ IL-6 receptor antagonist	Patients with lung cancer	Steroid refractory irAEs secondary to nivolumab showed relief	(101) Tocilizumab for the management of immune-mediated adverse events secondary to PD-1 blockade

However, prespecified results were not apparent in clinical trials by the use of single-agent PD-1/PD-L1 ICIs. The SARC028 trial evaluated the safety and efficacy of pembrolizumab and only 5% of patients with bone sarcoma (one OS and one chondrosarcoma) were observed to have an objective response (103). A novel phase 2 study (NCT03013127) validated the parallel result that pembrolizumab showed decent tolerance but insufficient clinical antitumor activity in adult patients with advanced OS (104). Further evidence on the lack of ICI activity in OS was also suggested with nivolumab (NCT02304458**)** and atezolizumab (NCT02541604) in pediatric trials. With 23 OS patients involved, neither study observed a positive response (90, 105). In addition, a trial (NCT03359018) of apatinib plus camrelizumab showed no extra survival benefits in the comparison of singe-agent apatinib (106). The low response in clinical trials with a single-agent ICI implied that OS was primarily resistant to the PD-1/PD-L1 blockade. The immune-genomic landscape of OS is characterized as a “cold” tumor, thus combination therapies of immune cell activation amplifiers synergized with ICIs were considered. In metastatic and orthotropic murine models of OS, bempegaldesleukin, a first-in-class CD122-preferred IL-2 pathway agonist, significantly enhanced activity of PD-1 and CTLA-4 ICIs through boosting the amassing of intratumoral effector T cells and NK cells (107). Another reason for the low objective response rate in PD-1/PD-L1 blockade therapy is that induced tumor-targeted CTLs are quickly exhausted in the tumor microenvironment so they cannot provide a sustainable and powerful anti-tumor effect (108). Consequently, it is apparent that single PD-1/PD-L1 ICI therapy may not be effective enough for treating OS. Lussier et al. revealed that T cells infiltrated in the OS tumor microenvironment unregulated additional inhibitory receptors like CTLA-4, which conspired to hinder tumor immunity. They combined CTLA-4 with a PD-1 ICI in the K7M2 murine model of metastatic OS and the tumors were completely under control in most of subjects as expected (109). Helm et al. found that OS mouse models treated with a combination of CTLA-4 and PD-1 ICIs detected a rise in CD8^+^ T cells. The Alliance A091401 trial aimed at evaluating nivolumab plus ipilimumab combination therapy for metastatic sarcoma and showed that in the combination group, 16% of patients reacted to the immunotherapy while only 5% of patients in the nivolumab group had a confirmed reaction (110). To date, although the therapeutic effect of ICI combination in OS has not been confirmed in clinical trials, several cases reported that immunotherapy with ipilimumab plus nivolumab displayed notable tumor manifestation remission and tumor mass stabilization with metastatic OS patients (111, 112). As is widely acknowledged, ICI-like anti-PD-1 inhibitors are antigen agnostic which directly points to a severe immune-related adverse event (irAE), immune hyperactivation. This cytokine release syndrome or cytokine storm can manifest as cardiovascular-associated and cerebrovascular-related events, coagulation disorders, encephalopathy, endocrinopathies, fever, gastro-hepatic-intestinal derangements, and hypoxia, and eventually progress to a stage of multiple organ failure. Steroids can be used to neutralize the toxicities, but they might impair the effectiveness of immunotherapy, and Cortellini et al. suggested that concomitant medication of a PD-1/PD-L1 ICI with prednisone can worsen patients’ ORR, PFS, and OS (113, 114). IL-6 blocker tocilizumab was tested to successfully temporize irAEs in a wide variety of models (115). It reminds us that a cytokine receptor antagonist plus ICI may be a therapeutic choice to manage a better prognosis in immunotherapy.

Interestingly, anti-PD-1 and anti-PD-L1 inhibitors have been found to mediate macrophages from M1 to M2 phenotypes in xenograft OS murine models (36). A characteristic of immune exclusion of OS pulmonary metastasis is the co-location of TAM and PD-L1-expressing tumor cells with CD8^+^ T cells on the pulmonary metastasis interface, posing a barrier to further TIL infiltration into pulmonary metastasis (69). Based on this, macrophage polarization-modulating drugs combined with a PD-1/PD-L1 ICI may help to break the barrier on the OS pulmonary metastasis interface and thus enhance the infiltration of TILs.

#### Novel Generation ICIs

It has been recognized that there are other checkpoint molecules co-expressed on TILs at the interface of tumors, conspiring against anti-tumor immunity. Among them, the checkpoint T-cell immunoglobulin and mucin-domain containing-3 (Tim-3) (72), lymphocyte-activation gene 3 (LAG-3) (116), indoleamine 2,3-dioxygenase (IDO) (69), and human epidermal growth factor receptor-2 (HER2) (117) have been examined in terms of OS.

Tim-3 regulates both the innate and adaptive immune pathways, and its expression has been confirmed in diverse immune cell types, involving CTLs, Tregs, macrophages, DCs, and NK cells (118, 119). The bond with its endogenous ligand GAL-9 leads to apoptosis and immune tolerance of T cells. A study indicated that Tim-3 was an independent predictor of survival, whose overexpression was a sign of poor prognosis in patients with OS (120). Furthermore, Tim-3 was proved to induce macrophages polarized to the M2 phenotype and promote lung metastasis in mouse models transplanted with OS-derived exosomes (121). In OS patients, both soluble Tim-3 and Tim-3-positive CD8^+^ and CD4^+^ T cells showed an elevated level in the peripheral circulation, which is negatively associated with the release of proinflammatory cytokines like IL-2, IFN-γ, and TNF-α (122). Similar to Tim-3, LAG-3 was broadly expressed on immune cells and inhibited anti-tumor immunity through interacting with ligand LSECtin in sarcoma (123). Pignon et al. suggested that cancerous patients with a higher percentage of CD8^+^PD-1^+^ T cells that are negative for Tim-3 and LAG-3 may better respond to anti-PD-1 immunotherapy (124). Assuming that there is a compensatory upregulation of Tim-3 and LAG-3 secondary to single-agent ICI therapy, resembling other diseases. Combinations of PD-1 ICI with Tim-3 or LAG-3 are rational candidates for further experiments in OS (116).

IDO is a tryptophan-degrading enzyme. The decrease in tryptophan and the rise in tryptophan metabolites restrain T-lymphocyte proliferation and induce immune tolerance. The activation of IDO can be induced by Bin1 gene losses and stimulation of IFN-γ in tumors. Especially, cytokines IL-12 and IL-18 also amplify IDO activity independently from the IFN-γ pathway in OS (125). Patients with higher IDO expression were associated with worse survival which may be attributable to the immune resistance induced by IDO (126). Toulmonde et al. revealed that TAM-expressing IDO1 is favorable to the M2 phenotype, indicating that a vital resistance mechanism to PD-1 ICI may be on account of preferential formation of these immune-suppressive phenotypic TAM (127). A clinical trial (NCT03414229) aimed at evaluating the combination of IDO inhibitor epacadostat with pembrolizumab in sarcoma is currently ongoing.

HER2 is highly expressed across a number of OS cell lines so is an ideal candidate for the combination of ICIs. Promisingly, a novel processing method that noncovalently binds TRA with nanomaterial grapheme oxide (GO) into stable TRA/GO complexes was achieved to eliminate OS cells, through inducing augmented oxidative stress and HER2 signaling (128). The clinical trial of TRA plus chemotherapy drug deruxtecan is ongoing (NCT04616560), and the cardiovascular safety in children and adolescent of this therapy has been proved in a previous study (129). We note that adjuvant high-dose chemotherapy is suggested to hinder tumor immune surveillance systems while killing tumor cells, and may increase the risk of a second malignancy as reported (130).

### Adoptive T-Cell Therapy (ACT)

ICIs serve to revive a suppressed or suboptimal immune system. On the contrary, ACT is another great promising immunotherapy which directly “tells” T cells the characterization of tumors, then selectively recognizes, orients, and attacks tumors. Over a few decades of intense studies, three main ACTs have been distinguished: chimeric antigen receptor (CAR)-engineered T cells, T cell receptor (TCR)-engineered T cells, and tumor-infiltrating lymphocytes (TILs) (131). This treatment strategy represented impressive clinical responses with ex vivo-manufactured cellular therapies aiming at tumor antigens. After CAR T cell therapy obtained extraordinary clinical success in hematologic malignancies and lymphoma with FDA approval, the FDA has granted *orphan drug* designation to a CLDN18.2-specific CAR T cell agent in gastric/GEJ cancer, indicating a breakthrough in the ACT of solid tumors (132).

#### CAR T Therapy

CAR T cells are engineered to recognize tumor-associated antigens (TAAs). Briefly, the transduced CAR consists of (i) an ectodomain derived from a single chain variable fragment of an antibody that recognizes a neoantigen, (ii) the transmembrane domain, and (iii) an endodomain with intracellular signaling domains derived from the CD3 ζ chain and co-stimulatory molecules. This structure enables T cells to recognize TAAs and result in T-cell-mediated tumor cytotoxicity in a major histocompatibility complex (MHC)-independent manner (133). CAR T cells targeting CD19 have shown an unprecedented response against B cell hematologic malignancies, reaching 90% remission rates in clinical trials and becoming the first genetically modified cell-based treatment approved by the FDA (134, 135).

Three FDA-approved CAR T cell products and virtually all the current clinical trials use γ-retroviral or lentiviral to modify gene ex vivo. Nevertheless, viral vectors come with some noteworthy safety concerns and production challenges. Due to the broad distribution of proviral insertion sites of retroviral vectors, oncogenic transformation may be secondary to potential insertional mutagenesis (131). Ruella et al. reported that a mis-insertion of the CAR gene into a single leukemic B cell mediated by lentiviral vector during T cell manufacturing can lead to overexpansion of CAR-transduced B cell leukemia (CARB) cells and irreversible death in a B cell acute lymphoblastic leukemia patient. Moreover, these CAR B cells integrate with CD19 on the surface of leukemic cells, disguising it from recognition by and conferring resistance to CAR T cells. Despite no exact mechanism being identified, leukemic blasts were detected to have lentiviral vector insertions in two sites: one was in chromosome 13 in intron 18 of the propionyl-CoA carboxylase-A gene9, and the other was in chromosome 11, 62.5 kb downstream of the neuropilin-1 gene. Although it is a specific case, the mis-insertion resulted in oncogenesis and resistance to CAR T cells. This study highlighted that we should be more vigilant, and that better manufacturing technologies are needed when using lentiviral vectors (136). In addition, the limitation of the long-lasting transgene expression induced by viral vectors may lead to persistent B cell aplasia and cytokine release syndrome (137, 138). In terms of production, lot-to-lot variation in viral transduction efficiencies as well as the high cost and time-consuming nature of the T cell manufacturing procedure also remain noteworthy challenges. Based on the defects above, novel non-viral transfection methods were designed and classified as membrane permeabilization-based means and carrier-based means, which are featured with an added bonus of high flexibility in effector molecules and cell types (131).

Due to the heterogeneity of tumor antigens, limited targets are identified in OS. Exhausted CAR T cells over-expressed inhibitive receptors such as PD-1, with an upregulation of PD-L1 on tumor cells at the same time. Combination therapy with PD-1 ICI or genetically engineered CAR T cells expressing tumor-limited PD-1 blockade can rescue the immunosuppression of single CAR T therapy partially (139). In addition, a clinical trial (NCT04433221) that combined CAR T cells with low dose chemotherapy which was demonstrated to modulate surface PD-L1 level is undergoing. Furthermore, multi-target CAR T cells are constructed to improve antigen recognition and avert tumor recurrence caused by the overgrowth of certain antigen-negative or low-expressed cells (140). In the view that CTLs co-expressing TIM-3-PD-1 function more exhaustedly than those expressing PD-1 only, homogenously inhibiting TIM-3 and PD-1 may better prime T cells and strengthen their anti-tumor cytotoxicity (141, 142). For instance, CD19 CAR T cells exhibiting considerable co-expression of inhibitory receptors TIM-3^+^/PD-1^+^ showed a lower CD4/CD8 ratio in TexMACS medium, indicating an increased immunity (143).

Another factor of failure in combating solid tumors with CAR T cells accounts for restricted infiltration and poor persistence caused by a stiff osteoid bone tumor matrix and immunosuppressive components in the TME (133). Hence the incorporation of costimulatory molecules and cytokines are designed to enhance CAR T-cell activation and strengthen its function. In fact, in order to improve costimulatory signaling, CD28-based and CD28-CD3ζ-OX40 CAR T cells are being tested in clinical trials respectively on patients with sarcoma (NCT00902044 and NCT01953900). The transgenic cytokine expression of IL-2, IL-15, or IL-23 on CAR T cells also improved proliferative activity (144). Simultaneously, T cells redirected for universal cytokine killing were developed with a nuclear factor of activated T cell (NFAT)-responsive promoter that only initiated cytokine release when CAR recognized a tumor antigen, making for minimizing systemic toxicity and elevating cytokine concentration at the tumor site (140). As to facilitating migration to the tumor site, CAR T cells were modified with co-expression of CCL5 and CXCL9 which created a loop to magnify lymphocyte engraftment through effective CD8^+^ recruitment (145).

#### TCR T Therapy

Different from CAR T cells, receptors expressed on the TCR T cells are produced from high affinity and high acidity tumor antigen-specific T-cell clones, which allow TCR T cells’ specificity and sensitivity for targeting cell-surface human leukocyte antigens (HLA) (146). Superior to antibodies and CARs in targeting efficiency, TCRs are able to penetrate tumors and engage with both tumor intracellular and surface antigenic peptides presented by HLA (147, 148). This feature allows for more latent targets and potential for further application in solid tumors. Cancer germline antigens express in a restricted manner in testis tissues and tumor tissues from different histological origins, while germ cells lack MHC molecule expression and are protected from TCR T cell-mediated immune attack (149). A cancer germline antigen NY-ESO-1 is an ideal target for TCR T cell therapy. In a phase I/II trial of NY-ESO-1-specific TCR-T treatment, 61% of synovial cell sarcoma patients benefited from the treatment without severe side effects (150). Moreover, NY-ESO-1 expression is found in 31.3% of OS tumors and TCR that targets NY-ESO-1 is being tested currently in patients with OS (NCT03462316). Most previous studies engineered CD8^+^ T cells that encode MHC-I-restricted TCRs for treatment, however, Lu et al. managed to treat patients with OS by CD4^+^ T cells transduced with MHC-II-restricted TCRs and MAGE-A3 and all experimented patients showed objective partial responses on metastatic lung lesions (151).

Papillomavirus binding factor (PBF) is a kind of DNA-binding transcription factor whose expression reaches up to 92% in OS. PBF A24.2 peptides are determined to activate CTLs from HLA-A24-positive patients with OS and therefore trigger immunity to eliminate tumor cells (152). A PBF TCR-multimer has been successfully created to recognize the naturally presented PBF peptide on HLA-A24^+^PBF^+^ OS cells (153). Although data about the usage of PBF-modified T cells in treating OS are not available, studies of this issue are critical, as suggested by the encouraging results with PBF’s role in oncogenicity and immunogenicity.

On the contrary, limitations of TCR T therapy come from the fact that tumors are likely to escape immunity by decreasing the expression of their MHCs and the usage is restricted in different patients with various HLA haplotypes because of HLA restriction (133). Baeuerle et al. solved this problem through developing TCR fusion constructs (TRuCs) with five fused TCR subunits, which activate anti-tumor immune response in a HLA-independent way. Of note, TRuC T cells showed better safety in well-controlled cytokine release compared with CAR T cell therapy (154). Another challenge is mispairing of the introduced α/β TCR chains with endogenous α/β TCR chains, that not only weakens the expression and efficacy of TCR T cells, but can also result in recognition of unintended antigens and may go further with autoimmune response and toxicities (101). A substitute of invariant natural killer T TCR T cells engineered and later differentiated from hematopoietic stem cells has been explored and potently protected a melanoma mouse model from lung metastasis (155). A relevant clinical trial in patients with OS is ongoing, testing the safety of transplanting TCRαβ^+^/CD19^+^-depleted haploidentical hematopoietic stem cells for treatment (NCT02508038).

#### TIL T Therapy

OS is characterized with a high proportion of TILs which play a vital role in regulating the progression of tumors. They were proposed with several advantages compared to non-infiltrating lymphocytes. First of all, TILs are organized from the TME with requisite chemokine receptors and thus they have highly specific directionality into tumors (156). Secondly, most TILs target mutated tumor-specific antigens instead of self-antigens, lowering the risk of autoimmunity subsequent to TIL T therapy (157). What is more, T cells recognizing mutated antigens is independent of central tolerance which allows for the expression of higher affinity TCR (158). Given those benefits, TIL T therapy was pioneered by Rosenberg in patients with melanoma, as the first adopted form of effective T cell therapy for solid tumors (159).

TILs are obtained from resected tumors followed by ex vivo expansion, and subsequently are transferred to patients in enormous quantity after lymphdepletion. Co-cultured with IL-2, TILs are exposed to rapid expansion protocols (REPs), under the restimulation of monoclonal anti-CD3 antibodies in the presence of allogeneic irradiated peripheral mononuclear cells and IL-2 (160). However, the isolation and expansion of TILs from OS tissue are considered to be uncertain, because the level of obtained TILs is far from sufficient regarding the requirement for immunotherapy (161). An additional obstacle in OS is low immunomodulatory molecules and suffusion of suppressive mediators on OS cells that may hinder activation and proliferation of TILs (162). The novel generation of TIL T cells showed satisfactory persistence, consistent with memory phenotypes of the majority of T cells. Sarnaik et al. reported a one-time cellular therapy named lifileucel (LN-144) with durable responses and an 80% disease control rate in advanced melanoma patients following failure of ICI therapy (163). The promising clinical trial about applying TIL LN-145 in treating patients with OS is ongoing (NCT03449108). Furthermore, combining ICI with TIL T cells may also represent a valid treatment option for OS patients progressing to individual therapies. Anti-CTLA-4 inhibitors were validated to boost HLA binding affinity of TIL T cells in melanoma as well as promoting CD8^+^ TILs expansion in Lewis lung carcinoma (164). The most recent study conducted by Wang et al. suggested that TILs plus anti-PD1 therapy showed remarkable clinical outcomes in metastatic OS patients, with a nearly quintuple objective response rate than single anti-PD1 therapy with a significantly prolonged medium progression-free survival time and medium overall survival time (165).

### Cancer Vaccines

Cancer vaccines are another novel immunotherapy that induce anti-tumor effects through stimulating patient’s endogenous immune response. Tumor antigens of whole cells, lysates, DNA, RNA, peptides, or proteins are presented or exposed to initiate the immune system (21, 166). In the 1970s, Marcove et al. pioneered the use of autologous tumor lysates as cancer vaccine therapy and resulted in increased overall survival in patients with OS (167). For cross-century researching, the usage of cancer vaccines has gained momentum and is mainly classified into three types: immune cell vaccines, autologous tumor cell vaccines, and non-cell-based vaccines. Immune cell vaccines were developed to exploit innate immunocyte (DCs, γδ T cells, and macrophages) advantages of activating effector T cells to the full. However, at the same time, limited by the immunosuppressive molecules in the TME and quality/quantity of compromised immune effector cells in patients, the availability of migration and activation is a main concern (66). On the other side, autologous tumor cell vaccines and non-cell-based vaccines bypass this obstacle. The recognition mechanism of autologous tumor cell vaccines is HLA-I-independent, and patient immune systems function to specifically select the most immunogenic antigen (164). Non-cell-based vaccines are characterized with higher efficiency and safety because these certain peptides or viruses can avoid an off-target effect (168).

#### Immune Cell Vaccines

DC vaccines are the most widely used vaccination approaches in tumors. DCs are professional antigen-presenting cells with robust stimulation function. They endocytose and present antigens to naïve T cells which are subsequently stimulated to differentiation into tumor-killing cells (66). DC vaccines are developed to reverse the tumor-induced inhibition of APC antigen presentation activity and remove immunosuppression (169, 170). The latest progress in DC vaccines is FDA-granted orphan drug designation for ilixadencel against hepatocellular carcinoma considering the great success obtained in a phase 1 trial implemented with ilixadencel in Sweden (171). The general manufacturing procedure of DC vaccines is as follows: DCs are isolated from peripheral blood mononuclear cells, matured and pulsed with tumor antigen ex vivo, and ultimately injected back into the patient (66). The classification based on the various sources of pulsed antigens is divided into three major types: 1) DCs co-cultured with tumor-specific peptides or proteins; 2) DCs transfected with DNA or RNA encoding for tumor antigens; and 3) DCs co-cultured with tumor lysates or fused with devitalized tumor cells. The first two approaches are particularly effective for identified antigenic targets, whereas the third one bypasses the necessity for identified antigens and automatically forces the patient’s immune system to target the most antineoplastic antigen (164). Krishnadas et al. showed a decent clinical response with DCs pulsed with peptides stemmed from cancer germline antigens (MAGE-A1, MAGE-A3, and NY-ESO-1) against neuroblastoma, Ewing sarcoma, osteosarcoma, and rhabdomyosarcoma (172). As for some kinds of OS with cancer germline antigen genes silenced, combining demethylating treatment can be introduced to raising their expression (173). Besides, combination therapy of DC vaccines and targeted drugs such as anti-transforming growth factor-β/glucocorticoid-induced tumor necrosis factor receptor antibodies, has been elucidated to efficiently suppress the progression of primary and metastatic tumors by remodeling the TME to be more immunized (174). Another notable improvement came from the source of DCs. The major source of DCs in clinical trials is human CD14^+^ monocytes or CD34^+^ progenitors, but lately Zhou et al. proved that type 1 conventional dendritic cells can also elicit systemic and sustaining tumor-specific T cell-mediated cytotoxicity in a mice model loaded with OS (175). This finding further expanded the usage and development of DC vaccines.

However, tumor-associated suppression results in some unsatisfactory outcomes in clinical trials of DC vaccines (172, 176). The combinations are being evaluated now with gemcitabine which inhibits myeloid-derived suppressor cells to improve effects of tumor-associated suppression (NCT01803152). To eliminate deregulation from immune checkpoints, ICIs are adopted together. For instance, Nagaoka et al. demonstrated that anti-PD-1 treatment enhanced the infiltration and function of TILs induced by DC vaccines in melanoma-loaded mice models (177). Blockade of CLTA-4 has also been reported to augment T cell priming capacity of the DC vaccine and showed reduced angiogenesis and metastasis progression in colon and breast tumor-bearing mice (178). Hence, using ICIs with DC vaccines plays a complementary role in elevating antitumor efficacy and warrants further evaluation in clinical trials.

Apart from DCs, another two types of immune cells, γδ T cells, and macrophages have been put forward for tumor vaccines in the form of peptide-pulsed γδ T vaccines and chimeric antigen receptor macrophages (CAR-Ms). γδ T cells have a powerful ability of priming CD8^+^ T cells (179). In addition, γδ T cells are superior to traditional DC vaccines because the way they activate cytotoxic activity against cancer cells is HLA-independent (66). In the initial study, the researchers reported that γδ T cells can directly recognize and attack OS cells, despite the fact that targeted cell lines were merely moderately susceptible to γδ T cell cytotoxicity (180). FDA-approved rapamycin as an mTOR inhibitor can enlarge the elicited γδ T cell response through boosting proinflammatory factor release and enhancing tumor core infiltration (181, 182). Given the intrinsic function to penetrate tumors and activate T cell-mediated anti-tumor immunity, researchers recently engineered novel CAR-Ms to attain three aims: 1) presenting tumor antigens to naïve T cells and instigating them into CTLs, 2) fostering a pro-inflammatory TME by converting M2 macrophages to the M1 phenotype, and 3) phagocytizing tumor cells directly. CAR-Ms can be endowed with stable M1 phenotype after being genetically engineered by the chimeric adenoviral vector (Ad5f35). Klichinsky et al. developed an anti-HER2 CAR-M which displayed a notable reduction in tumor burden and a prolonged overall survival in mice models with metastatic lung cancer (183). To optimize the producing process and elude the risk of viral vectors’ oncogenicity with ex vivo CAR-M production, Kang et al. fabricated polymer nanocarriers which delivered genes encoding CAR and interferon-γ to macrophages *in vivo* and managed to *in situ* code to tumor-specific CAR-expressing and M1 phenotype macrophages (184). Thus far, these previous studies may pave the way for applying γδ T cell- and CAR-M-based vaccines in treating OS.

#### Autologous Tumor Cell Vaccines

Autologous tumor cell vaccines bypass DC isolation and culture ex vivo, and directly initiate DC response *in vivo*. Tumor cells are isolated from the patient, expanded if necessary, and then receive irradiation before being re-fused into the patient (185). In patients with EWS, an autologous tumor cell vaccine pulsed with GM-CSF and a furin convertase-targeted snRNA has been demonstrated to trigger a valid immune response in half of the patients (186). In the field of OS, a recent study combined an autologous tumor cell vaccine, ACT, and IL-2 together in dogs with OS and obtained remarkably prolonged survival compared to traditional amputation therapy (187). Apparently an autologous tumor cell vaccine has great potential in single or combination therapy, but its safety and efficacy in human beings with OS requires further study to elucidate.

#### Non-Cell-Based Vaccine

New approaches are breathing new life to tumor vaccine strategies. Peptide- and viral-based vaccines have a similar mechanism of presenting the antigen directly to DCs *in vivo*. The tumor–associated antigen PBF-derived peptide vaccination has long been explored for HLA-A2/A24^+^ patients with OS, Li et al. performed a breakthrough study in creating a peptide-specific tetramer targeting QVT and LSA peptides and showed cytotoxicity against HLA-A11^+^PBF^+^ OS cells (188). Other vaccinations based on HER2 have been underscored by benefits in canine models with OS, including a HER2-targeted recombinant listeria vaccine and an epidermal growth factor receptor (homology to HER2)-targeted peptide vaccine, which reduced metastasis and improved prognosis compared to the control group (189, 190). The peptide-based vaccine has to function in the context of HLA-I, and immunologic adjuvant is needed to gain a sufficient T cell response. Apart from polarization-inducing ability on macrophages mentioned above, mifamurtide can stimulate TLR4 to upregulate the expression of type 1 interferon, so it may have the potential as a substitute for INF-α (164). Furthermore, two recently completed phase I/II clinical trials with oncolytic HSV1716 (NCT00931931) and unmodified oncolytic reovirus REOLYSIN^®^ (NCT00503295) explored their effectiveness in treating OS, which may become novel approaches for oncolytic viral vaccines.

## Conclusion

Based on deepening understanding of the biological characteristics of OS, treatment has broadly developed across the centuries. Immunotherapy has revolutionized the management of OS since it was introduced, which can change non-responders to responders and strengthen the responses that do occur. In this review, we explored the specific TME and current application of immunotherapy in OS, classified as ICIs, ACT, and cancer vaccines. OS induces an immune-negative microenvironment characterized by high densities of M2 macrophages and low densities of TILs, which leads to drug resistance and inferior overall survival of OS patients. Particularly, immunotherapy breaks bottlenecks in tumor immune escape and chemotherapeutic resistance; ICIs target those protein molecules or their ligands that downregulate autoimmune function and thus re-activate the autoimmune system. ACT produces engineered T lymphocytes with high precision and efficiency which are equipped with specific neoplastic antigen-targeted receptors *in vitro*. Tumor vaccines are a range of biological ornaments loaded with TAA to provoke the immune system into recognizing and attacking those extrinsic antigens. Immunotherapy in melanoma, prostate cancer, renal cell carcinoma, and non-small-cell lung cancer has become a reality, and is now approved by the FDA.

However, to date, novel immunotherapies remain limited for OS. The main obstacles focus on two fields: finite T-cell infiltration and secondary immune toxicity. It is hard to permeate into targeted tissues because of immunosuppressive TME and dense fibrous tissue around solid tumors that impede T-cell infiltration. Oncolytic virus, which can infect and lyse tumor cells, has been identified to reverse the primary resistance to PD-1 blockade therapy through increasing intratumoral infiltration by CD8+ T cells and elevating PD-L1 expression (191, 192). At present, oncolytic virus for OS is in preclinical exploration and it is hoped that it can break the defense of solid surfaces in OS (193). Moreover, angiotensin inhibitors have been suggested to lessen extracellular matrix sclerosis in solid tumors, involving pancreatic tumors, breast tumors, and colorectal adenocarcinoma (194, 195). In addition, nanotechnology has become widespread in medication. These biodegradable nanoparticles are used as adjuvants to guide and control molecules that come into play at targeted sites (196). They can enter certain cells through phagocytosis, specific endocytosis, or by penetrating cells directly and thus induce a series of inflammatory chemokines to be released in the TME, and eventually attract T-cell infiltration (197). Beyond enhancing delivery, biomaterials can be applied in prompting the expansion of T cells ex vivo, such as constructing an APC-mimetic scaffold to precisely reproduce expansion stimulus signals *in vivo* (198). Multitarget approaches, providing a simultaneous inhibition of TME components, are being considered to offer a more efficient way to treat cancer. At present, the “immune cocktail therapy” of combining multiple immunotherapeutic strategies holds great promise, which can modulate the cancer-immunity cycle including but not limited to increasing immune cell infiltration and augmenting the cytotoxicity of T cells. We summarize the ongoing or planned clinical experiments that were mentioned above in **Table 3**.

**Table 3:** Some ongoing or planned clinical trials of novel immunotherapy.

Agent	Approach	Cancer	Phase	NCT number	Status
Nivolumab+ ipilimumab	PD-1 inhibitor+ CTLA-4 inhibitor	Metastatic melanoma Recurrent Ewing sarcoma/peripheral primitive neuroectodermal tumor Recurrent Hodgkin lymphoma Recurrent malignant solid neoplasm Recurrent melanoma Recurrent neuroblastoma Recurrent non-Hodgkin lymphoma Recurrent osteosarcoma Recurrent rhabdomyosarcoma Refractory Ewing sarcoma/peripheral primitive neuroectodermal tumor Refractory Hodgkin lymphoma Refractory malignant solid neoplasm Refractory melanoma Refractory neuroblastoma Refractory non-Hodgkin lymphoma Refractory osteosarcoma Refractory rhabdomyosarcoma Stage III cutaneous melanoma AJCC v7 Stage IIIA cutaneous melanoma AJCC v7 Stage IIIB cutaneous melanoma AJCC v7 Stage IIIC cutaneous melanoma AJCC v7 Stage IV cutaneous melanoma AJCC v6 and v7 Unresectable melanoma	I+ II	NCT02304458	Active, not recruiting
Pembrolizumab+ epacadostat	PD-1 inhibitor+ IDO1 inhibitor	Sarcoma	II	NCT03414229	Active, not recruiting
Trastuzumab + deruxtecan	HER-2 inhibitor+ chemotherapy	Osteosarcoma Recurrent osteosarcoma	II	NCT04616560	Recruiting
Unknown	CAR-T cells+ tumor vaccine	Sarcoma Osteoid sarcoma Ewing sarcoma	I+ II	NCT04433221	Recruiting
B7H3-CAR-T cells B7H3-CD19-CAR-T cells	CAR-T cells	Pediatric solid tumor Germ cell tumor Retinoblastoma Hepatoblastoma Wilms tumor Rhabdoid tumor carcinoma Osteosarcoma Ewing sarcoma Rhabdomyosarcoma Synovial sarcoma Clear cell sarcoma Malignant peripheral nerve sheath tumor Desmoplastic small round cell tumor Soft tissue sarcoma Neuroblastoma Melanoma	I	NCT04483778	Recruiting
HER2-CAR-T cells+ fludarabine+ cyclophosphamide	CAR-T cells	Soft tissue sarcoma	I	NCT00902044	Active, not recruiting
iC9-GD2-CAR-VZV-CTL+ fludarabine+ cyclophosphamide	CAR-T cells	Osteosarcoma Neuroblastoma	I	NCT01953900	Active, not recruiting
NY-ESO-1 TCR cells	TCR-T cells	Bone Sarcoma Soft tissue sarcoma	I	NCT03462316	Recruiting
TCRαβ+/CD19+ depleted haploidentical HSCT + zoledronate	TCR-T cells	Acute myeloid leukemia Acute lymphoblastic leukemia Hodgkin lymphoma Non-Hodgkin lymphoma Myelodysplastic syndrome Myeloproliferative syndrome Rhabdomyosarcoma Ewing sarcoma Primitive neuroectodermal tumor Osteosarcoma Neuroblastoma	I	NCT02508038	Recruiting
LN-145/LN-145-S1 TIL+ nivolumab+ ipilimumab+ fludarabine+ cyclophosphamide	TIL T cells+ PD-1 inhibitor+ CTLA-4 inhibitor	Bone sarcoma Dedifferentiated chondrosarcoma Giant cell tumor of bone Malignancy in giant cell tumor of bone Malignant solid neoplasm Ovarian carcinosarcoma Platinum-resistant ovarian carcinoma Poorly differentiated thyroid gland carcinoma Recurrent osteosarcoma Recurrent ovarian carcinoma Refractory osteosarcoma Soft tissue sarcoma Thyroid gland anaplastic carcinoma Thyroid gland squamous cell carcinoma Undifferentiated high-grade pleomorphic sarcoma of bone	II	NCT03449108	Recruiting
DC vaccine+ gemcitabine+ imiquimod	DC vaccine	Soft tissue sarcoma Bone sarcoma	I	NCT01803152	Active, not recruiting

With regard to toxicity, immune agents can cause systematic cytokine release syndrome, capillary leak syndrome, and a sepsis-like syndrome, and irAEs can be involved in any organ or tissue. It has been reported that combination of ICIs brought about more severe and broader AEs and worse even, irAEs not only appear in the early stage of treatment but also may develop in a prolonged period (199). To lower the risk of side effects to the maximum extent, more personalized therapeutic approaches are in urgent need of development. Patients should be stratified by the susceptibility to develop irAEs before starting, with the identification of biomarkers that predict patient responses and control the progress of treatment. In addition, agents need to be altered on the basis of targets recognized in patient biopsy samples and usability immune-related grading in clinical trials. In general, immunotherapy poses great prospects for the treatment of OS and further research is needed on molecular mechanisms to realize more accurate curative effects.

## Author Contributions

The co-first author is responsible for paper writing and literature search. The co-corresponding author is responsible for proofreading the article. The other authors are all members of this research group, and they jointly undertake the drawing and revision work of the paper. All authors contributed to the article and approved the submitted version.

## Funding

Science and Technology Project of Gansu Province (20JR5RA325) and Lanzhou Science and Technology Bureau Project (2017-4-60).

## Conflict of Interest

The authors declare that the research was conducted in the absence of any commercial or financial relationships that could be construed as a potential conflict of interest.

## Publisher’s Note

All claims expressed in this article are solely those of the authors and do not necessarily represent those of their affiliated organizations, or those of the publisher, the editors and the reviewers. Any product that may be evaluated in this article, or claim that may be made by its manufacturer, is not guaranteed or endorsed by the publisher.

## Abbreviations

OS, osteosarcoma; MSCs, mesenchymal stem cells; DCs, dendritic cells; NK, natural killer; TAMs, tumor-associated macrophages; iNOS, inducible nitric-oxide synthase; EMA, European Medical Agency; ATRA, all-trans retinoic acid; CSCs, cancer stem cells; CAR-Ms, chimeric antigen receptor macrophages; FDA, Food and Drug Administration; PHNPs, porous hollow iron oxide nanoparticles; TILs, tumor-infiltrating lymphocytes; Tfh, follicular helper T; PD-1, programmed cell death protein 1; CTLA-4, cytotoxic T-lymphocyte associated protein 4; irAE, immune-related adverse event; Tim-3, T-cell immunoglobulin and mucin-domain containing-3; LAG-3, lymphocyte-activation gene 3; IDO, indoleamine 2,3-dioxygenase; HER2, human epidermal growth factor receptor-2; TRA, trastuzumab; GO, grapheme oxide; ACT, adoptive T-cell therapy; CAR, chimeric antigen receptor; TCR, T cell receptor; TILs, tumor-infiltrating lymphocytes; TAAs, tumor-associated antigens; MHC, major histocompatibility complex; CARB, CAR-transduced B cell leukemia; NFAT, nuclear factor of activated T cells; HLA, human leukocyte antigens; PBF, papillomavirus binding factor; TRuCs, TCR fusion constructs; REPs, rapid expansion protocols.
